# Distinct features of hypereosinophilic syndrome with neuropathy from eosinophilic granulomatosis with polyangiitis

**DOI:** 10.3389/fneur.2022.1057767

**Published:** 2022-11-15

**Authors:** Hiroki Takeuchi, Kazuyuki Kawamura, Teruaki Kawasaki, Nobuyuki Oka

**Affiliations:** ^1^Department of Neurology, National Hospital Organization Minami Kyoto Hospital, Kyoto, Japan; ^2^Kyoto Clinical and Translational Research Center for Neurocognitive Disorders, Kyoto, Japan; ^3^Department of Neurology, Kyoto Konoe Rehabilitation Hospital, Kyoto, Japan

**Keywords:** hypereosinophilic syndrome (HES), eosinophilic granulomatosis with polyangiitis, vasculitis, peripheral neuropathy (PN), neuropathology

## Abstract

**Background and objectives:**

Hypereosinophilic syndrome (HES) and eosinophilic granulomatosis with polyangiitis (EGPA) have overlapping clinical considerations, which frequently involve peripheral neuropathy. The current study aimed to discriminate between the clinicopathological features of HES and EGPA, focusing on the mechanism of peripheral nerve damage.

**Methods:**

A total of 53 patients who underwent nerve biopsies at our laboratory were examined: nine patients with idiopathic HES (iHES), three patients with reactive HES, 14 patients with myeloperoxidase-anti-neutrophil cytoplasmic antibody (ANCA)-positive EGPA, and 27 patients with negative EGPA. Nerve biopsies were performed using light and electron microscopy.

**Results:**

Polyneuropathy was more common than mononeuritis multiplex in iHES, which differed from that in ANCA-negative EGPA groups (*p* = 0.012). Nerve biopsies showed that iHES was associated with neuropathy features such as rare vasculitis and non-vasculitic eosinophilic infiltrates, which differed from those of ANCA-negative EGPA. Fibrinoid necrosis was found only in the reactive HES and ANCA-positive groups. The percentage of endoneurial vessels occluded with eosinophils tended to be higher in iHES (1.8%) than in ANCA-positive EGPA (0%) and negative EGPA (0.7%). In a patient with ANCA-negative EGPA, the endoneurial vessels were occluded with platelets, fibrinoid materials, and eosinophils, demonstrating the morphology of eosinophil extracellular traps.

**Conclusion:**

iHES with neuropathy showed a pattern more similar to polyneuropathy than mononeuritis multiplex, which is dominant in ANCA-negative EGPA, and tended to show vasculitis in the peripheral nerves less frequently compared with EGPA. Eosinophilic infiltration and endoneurial vascular occlusion by eosinophils may cause nerve damage.

## Introduction

Systemic vasculitis involves various organs, with anti-neutrophil cytoplasmic antibody (ANCA)-associated vasculitis being the most frequent cause of peripheral neuropathy (1). In particular, eosinophilic granulomatosis with polyangiitis (EGPA) typically presents as adult-onset asthma and increased eosinophils in the blood and tissues. Peripheral nerves are the most frequently involved organs (2, 3), and despite the advancements in immunoregulatory therapy, neuropathy remains one of the serious symptoms of EGPA.

Although EGPA has been classified as an ANCA-associated vasculitis, only 30%−35% of patients are positive for ANCA (4). Both vessel inflammation and eosinophilic tissue infiltration have been proposed as causes of organ damage. The presence or absence of ANCA may be used to establish subgroups considering that renal involvement is more common in patients who are ANCA-positive, whereas cardiac disease is more frequent among those who are ANCA-negative (5–7); however, neuropathy occurs in both subgroups. Our previous report demonstrated the pathological differences between these subgroups following nerve biopsy, showing that necrotizing vasculitis was more frequent in patients who are ANCA-positive; whereas massive eosinophilic infiltration and eosinophilic degranulation in the endoneurium were observed only in those who are ANCA-negative (8). However, the respective mechanisms for these differences are not well understood (9). Interestingly, the clinicopathological difference between the subtypes illustrates that epineurial vessel occlusion by the intraluminal eosinophils may play an important role in pathology in patients who are ANCA-negative (10). Moreover, ANCA can activate neutrophils to release cytokines with filamentous DNA that induces cell death, a process termed NETosis (11). Recently, eosinophil extracellular traps have also been reported in allergic diseases, possibly contributing to microthrombi in EGPA (12, 13), which may induce ischemic processes in nerve tissues.

Hypereosinophilic syndrome (HES) is defined based on organ damage attributed to eosinophilic infiltration, with unexplained blood eosinophilia >1,500/μl (14– 16). In clinical practice, HES is often difficult to differentiate from EGPA, given that the allergic components are common to both (17), with HES occasionally presenting with peripheral neuropathy. However, considering that asthma and serum ANCA are negative in HES, EGPA and HES are possibly distinct disorders that require distinguishment for better therapeutic outcomes. A recent study showed that HES also showed biopsy-confirmed vasculitis involving the skin, colon, and lungs, and was addressed as HES-related vasculitis (18). However, peripheral nerve biopsy-confirmed eosinophilic vasculitis in HES seems rare (19, 20). Although C-reactive protein (CRP) levels have been reported to be lower in HES than in EGPA (21), there are lack of validated biomarkers in HES (22). Recently, HES has been divided into several categories, namely myeloproliferative, associated, and undefined HES (15). We targeted patients with HES who have no obvious cause for hypereosinophilia (idiopathic HES; iHES); given the importance of distinguishing between iHES and EGPA, HES associated with infection or lymphoma was studied secondarily. The current study therefore aimed to clarify the distinct characteristics of HES-related neuropathy, focusing especially on ANCA-negative EGPA with neuropathy, using clinicopathological methods, since the absence of ANCA makes a differential diagnosis difficult.

## Materials and methods

Patients referred to our laboratory for nerve biopsies in the last 22 years were retrospectively reviewed. We identified 12 patients with HES-associated neuropathy after exclusion of EGPA. Three of the 12 cases were considered reactive HES as described later. HES was defined based on organ damage attributed to tissue eosinophilia, with the peripheral nervous system appearing to be the target organ. This was diagnosed according to the criteria described in a previous study (21). In general, the patients had neither asthma nor para sinusitis, and had blood eosinophilia >1,500/μl with no obvious causes. The classical criteria for HES (14, 16) requires persistent (≥6 months) eosinophilia. In modern practice, however, the various therapies modify this. Thus, in the current study, hypereosinophilia is defined based on an eosinophil count of ≥1,500/μl before treatment. After excluding patients with myeloproliferative/neoplastic diseases, four of the nine cases underwent bone marrow examination. All cases exhibited peripheral neuropathy, with nerve biopsy showing various degrees of nerve damage that was unexplained by other causes. We identified three other neuropathic patients with hypereosinophilia-associated etiology (16) (one with hepatitis B, one with human T-lymphotropic virus type 1, and one with lymphocytic lymphoma). These patients were included in the study as reactive HES cases.

For the comparison with EGPA, 41 consecutive patients with EGPA who had neuropathy in the same period were studied. The number of myeloperoxidase-ANCA-positive or negative patients was 14 and 27, respectively. EGPA was diagnosed according to the criteria as previously described (23). Additionally, routine laboratory tests, underlying systemic disorders, and nerve conduction studies were also examined. Patients who had other causes of neuropathy or had already initiated immunotherapy for vasculitis before the biopsy were excluded.

The pattern of neuropathy (mononeuritis multiplex or polyneuropathy) was assessed using the distribution and progression pattern of neurological deficits from onset until the biopsy. Patients possessing multiple individual nerves with asymmetrical and stepwise progression of neuropathic symptoms indicate mononeuritis multiplex. The results of the nerve conduction study were also considered.

### Standard protocol approval, registration, and patient consent

This study conformed with the Ethical Guidelines for Medical and Health Research Involving Human Subjects established by the Japanese government and was approved by the National Hospital Organization Minami Kyoto Hospital (2021-20). Given the retrospective nature of our analysis, the ethics board determined that participant consent was not required. Nevertheless, written informed consent was obtained from all the participants for diagnostic nerve biopsy.

### Pathological methods

Biopsies from the sural nerves were assessed for the following parameters using standard EPON-embedded transverse sections stained with toluidine-blue and hematoxylin-eosin-stained paraffin sections (24): eosinophilic vasculitis, necrotizing vasculitis, epineurial eosinophilic infiltration, intraluminal eosinophils of the epineurial or endoneurial vessels, endoneurial infiltration of eosinophils, and degree of nerve degeneration. Peripheral nerve vasculitis was diagnosed according to the criteria of pathologically definite or probable vasculitic neuropathy (25). In each case, several sections of EPON-embedded samples cut at least 5 mm apart from each other were examined to identify vasculitis. The intraluminal space occupied by eosinophils was analyzed using ImageJ software Ver 1.53k (National Institutes of Health, Bethesda, MD, USA) (10) using EPON sections. Patients with >50% of their vessel lumens occupied by eosinophils were counted. Regarding epineurial eosinophilic infiltration, patients were divided into two groups according to the number of extravascular eosinophils (i.e., >50 or <50).

The proportion of axonal degeneration was examined using the teased nerve fiber technique. Focal myelinated fiber loss was identified by two observers using the toluidine-blue stained sections. Myelinated fiber density was counted in each nerve fascicle. The middle value in all nerve fascicles was obtained as the myelinated fiber density of the patient.

Selected samples were studied under an electron microscope (Hitachi-7650, Tokyo, Japan), especially the eosinophils and degranulation of eosinophils in the endoneurium.

### Statistical analyses

Quantitative variables were analyzed using the Mann–Whitney *U*-test or Kruskal–Wallis test, whereas categorical variables were compared using Fisher's exact probability test. All statistical analyses were performed using EZR (Saitama Medical Center, Jichi Medical University, Saitama, Japan).

## Results

### Patient characteristics

Patient characteristics were shown in Table 1. iHES-related neuropathy was observed in five men and four women. The age at biopsy was 63.1 ± 16.6 years (mean ± standard deviation). ANCA was negative. Associated organ involvement included the lungs in four cases, skin in five, gastrointestinal tract in one, and mastitis in one. Polyneuropathy (*n* = 6) was a more common pattern of neuropathy than mononeuritis multiplex (*n* = 3).

**Table 1:** Patient characteristics.

	**HES** **(*n* = 9)**	**ANCA + EGPA** **(*n* = 14)**	**ANCA-EGPA** **(*n* = 27)**	***p*-Value (Kruskal–Wallis or Fisher exact test among 3 groups)**	***p*-Value between 2 groups**
Sex (M/F)	5/4	6/8	14/13	0.86	
Age (year)	63.1 ± 16.6	66.8 ± 9.3	62.9 ± 11.2	0.59	
Asthma/parasinusitis	0/0	13/1	26/4		
Involved organs	Lung: 4 Skin: 5 Gastrointestinal: 1 Mastitis: 1	Lung: 4 Skin: 6 Gastrointestinal: 1	Lung: 3 Skin: 6 Heart: 2		
**Pattern of neuropathy**
Mononeuritis multiplex	3	9	22	0.022	HES vs. vs. NEGPA: 0.012
Polyneuropathy	6	5	5		
Sensory disturbance	7 (78%)	12 (86%)	25 (93%)	0.52	
Muscle weakness	7 (78%)	12 (86%)	19 (70%)	0.68	
Time from onset to biopsy (months)	21 ± 1.6	1.3 ± 0.9	1.8 ± 1.3	0.38	
CRP (mg/dl)	6.8 ± 9.8	2.9 ± 2.7	3.5 ± 4.6	0.85	
Eosinophils (mm^3^)	11,380 ± 11,270	9,370 ± 6,380	7,270 ± 6,021	0.62	
Decreased CMAP (tibial nerve) (*N*)	9/9 (100%)	8/11 (73%)	21/22 (95.4%)	0.3	

ANCA, anti-neutrophil cytoplasmic antibodies; CMAP, compound muscle action potential; CRP, C-reactive protein; EGPA, eosinophilic granulomatosis with polyangiitis; F, female; HES, hypereosinophilic syndrome; M, male; NEGPA, ANCA-negative EGPA; SD, standard deviation.

Data are shown as numbers (percent) or mean ± SD.

Statistical analyses were performed using the Mann–Whitney U or Kruskal–Wallis test for quantitative variables or Fisher's exact probability test for categorial variables.

We identified three patients with reactive HES, all of whom exhibited polyneuropathy. Few cases had underlying disorders: two with viral infections (hepatitis B and human T-lymphotropic virus type 1 carrier) and one with lymphocytic lymphoma.

Among patients with EGPA, 14 and 27 were myeloperoxidase-ANCA-positive and -negative, respectively. No significant differences in sex, age, and duration from onset to biopsy were observed among patients with iHES, ANCA-positive EGPA, and ANCA-negative EGPA. Although no differences in the frequency of sensory and motor symptoms were observed, differences in the pattern of neuropathy (mononeuritis multiplex/polyneuropathy) were observed between the iHES and ANCA-negative EGPA groups (*p* = 0.012). Mononeuritis multiplex was common in EGPA.

### Laboratory findings

No differences in CRP and number of blood eosinophils were observed among the iHES, ANCA-positive EGPA, and ANCA-negative EGPA groups.

### Nerve biopsy

Results of statistical analysis of nerve biopsy findings were listed in Table 2, and representative findings were shown in each figure.

**Table 2:** Findings of nerve biopsies.

	**HES** **(*n* = 9)**	**ANCA + EGPA** **(*n* = 14)**	**ANCA-EGPA** **(*n* = 27)**	***p*-Value (Kruskal–Wallis or Fisher exact test among 3 groups)**	***p*-Value between 2 groups**
Myelinated fiber density (mm^2^)	2,074 ± 2,066	3,651 ± 1,633	2,324 ± 1,616	0.025	HES vs. PEGPA: 0.039
					NEGPA vs. PEGPA: 0.015
Axonal degeneration (%)	64.1 ± 27.9	52.8 ± 35.0	71.9 ± 26.5	0.19	
Focal myelinated fiber loss	2 (22%)	5 (36%)	13 (47%)	0.36	
Epineurial vessel destruction	1 (11%)	9 (64%)	14 (52%)	0.0355	HES vs. PEGPA: 0.029
					HES vs. NEGPA: 0.051
Fibrinoid necrosis of epineurial vessel	0	6 (43%)	0	0.0003	
Total no. of epineurial vessels	43.5 ± 12.4	36.6 ± 14.2	42.5 ± 10.1	0.062	
Eosinophils in epineurium (>50/50>)/No. of cases	5/4	1/13	11/16	0.026	HES vs. PEGPA: 0.018
					PEGPA vs. NEGPA: 0.033
% of epineurial vessels occluded by eosinophils	0.39 ± 1.17	0.86 ± 1.56	2.14 ± 3.13	0.23	HES vs. NEGPA: 0.11
Extravascular eosinophils in endoneurium (no. of cases, %)	2 (22%)	3 (21%)	10 (37%)	0.59	
No. of Eo/No. of nerve fascicles	0.34 ± 0.69	0.02 ± 0.07	0.17 ± 0.39	0.21	HES vs. PEGPA: 0.09
					PEGPA vs. NEGPA: 0.15
% of endoneurial vessels occluded by eosinophils	1.76 ± 3.49	0	0.66 ± 2.37	0.19	HES vs. PEGPA: 0.07
					HES vs. NEGPA: 0.11

ANCA, anti-neutrophil cytoplasmic antibodies; EGPA, eosinophilic granulomatosis with polyangiitis; Eo, Eosinophils; HES, hypereosinophilic syndrome; NEGPA, ANCA-negative EGPA; PEGPA, ANCA-positive EGPA. Data are shown as numbers (percent) or mean ± SD.

Statistical analyses were performed using the Mann–Whitney U or Kruskal–Wallis test for quantitative variables or Fisher's exact probability test for categorial variables.

#### Myelinated nerve fiber loss

**Figure 1:**
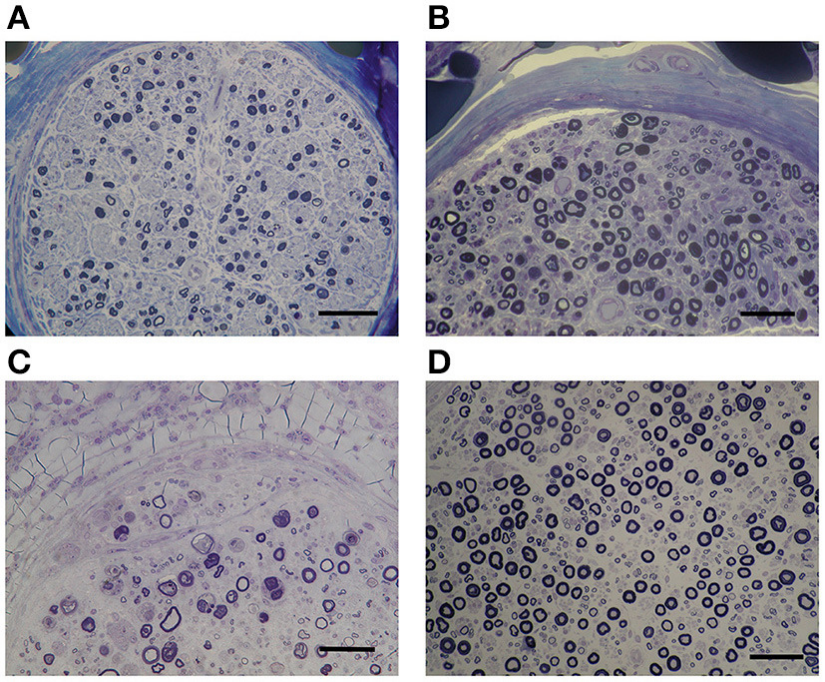
Nerve degeneration and myelinated nerve fiber loss. EPON-embedded section with toluidine-blue staining in a case of idiopathic hypereosinophilic syndrome (iHES) **(A)** ANCA-positive EGPA **(B)** and ANCA-negative EGPA**(C)**. Myelinated fiber density was better preserved in the ANCA-positive EGPA sample **(B)** than th in iHES **(A)** or ANCA-negative EGPA **(C)**. No differences in the ratios of axonal degeneration were observed among all three groups. A normal nerve is shown for reference **(D)**. ANCA, anti-neutrophil cytoplasmic antibodies; EGPA, eosinophilic granulomatosis with polyangiitis.

Nerve biopsy indicated varying degrees of axonal degeneration. Myelinated fiber density was more preserved in the ANCA-positive EGPA than that in iHES or ANCA-negative EGPA groups (*p* = 0.025). However, no differences in the ratios of axonal degeneration were observed among all three groups. A 22% focal myelinated fiber loss was observed in patients with iHES, with most of them exhibiting diffuse axonal degeneration (Figure 1A). No significant difference in the frequency of focal fiber loss was observed among the three groups (Table 2, Figures 1B,C). A nerve with normal appearance is shown for reference (Figure 1D).

### Vasculitis features

**Figure 2:**
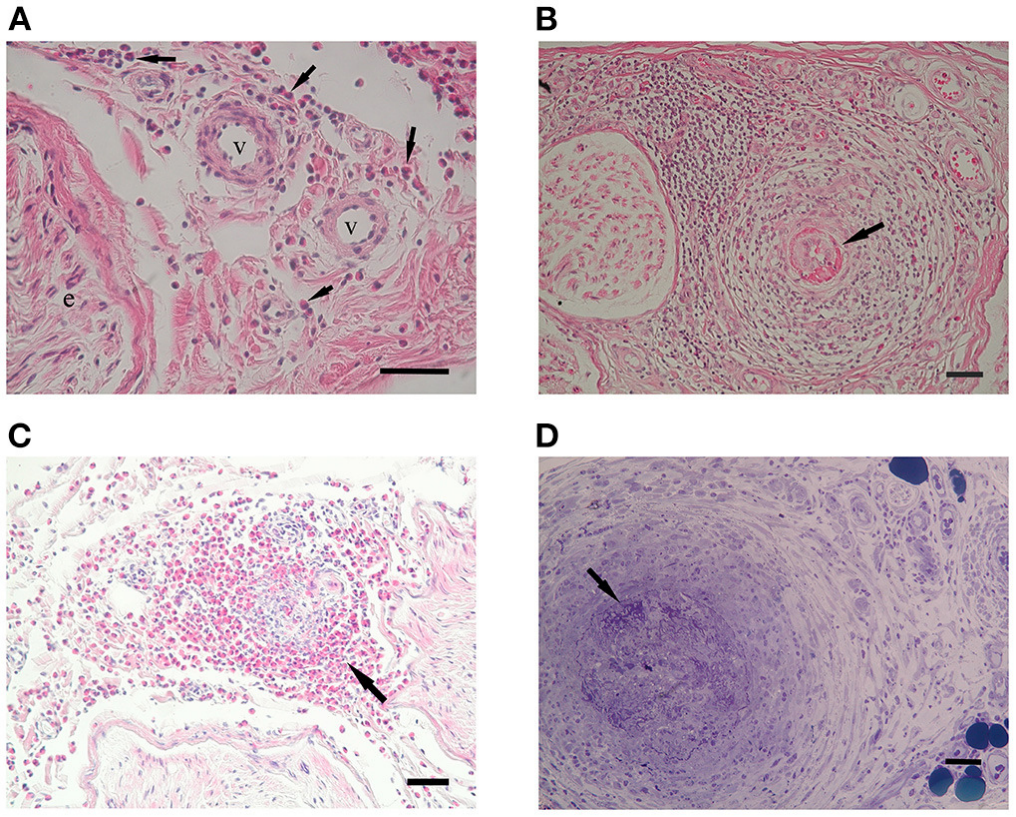
Vasculitic features. Hematoxylin and eosin (HE) staining in patients with iHES **(A)** ANCA-positive EGPA **(B)** and ANCA-negative EGPA **(C)**. In iHES, epineurial vessel walls show no disruption, although infiltrates of eosinophils (arrows) can be observed in the perivascular interspace. In ANCA-positive EGPA, fibrinoid necrosis of the endothelial layer (arrow) and infiltrates in the vessel wall are visible. In ANCA-negative EGPA, epineurial vasculitis with massive eosinophilic infiltrates are visible, although no fibrinoid necrosis can be seen. **(D)** A patient with reactive HES showed fibrinoid necrosis and small arteritis. Scale bar: 50 μm for **(A–D)**. ANCA, anti-neutrophil cytoplasmic antibodies; EGPA, eosinophilic granulomatosis with polyangiitis; iHES, idiopathic hypereosinophilic syndrome.

No difference in the total number of epineurial vessels was observed among the three groups. Epineurial vessel destruction was rare in the iHES group (11%) (Figure 2A), which was significantly lesser than that in ANCA-positive EGPA (Figure 2B; *p* = 0.029), and tended to be lower than that in the ANCA-negative EGPA (*p* = 0.051; Figure 2C). Fibrinoid necrosis was not observed in the iHES and ANCA-negative EGPA groups, although was observed in 43% of patients with ANCA-positive EGPA (*p* = 0.0003). However, two of the three patients with reactive HES showed fibrinoid necrosis in small arteries and arterioles (Figure 2D).

### Eosinophilic infiltration

**Figure 3:**
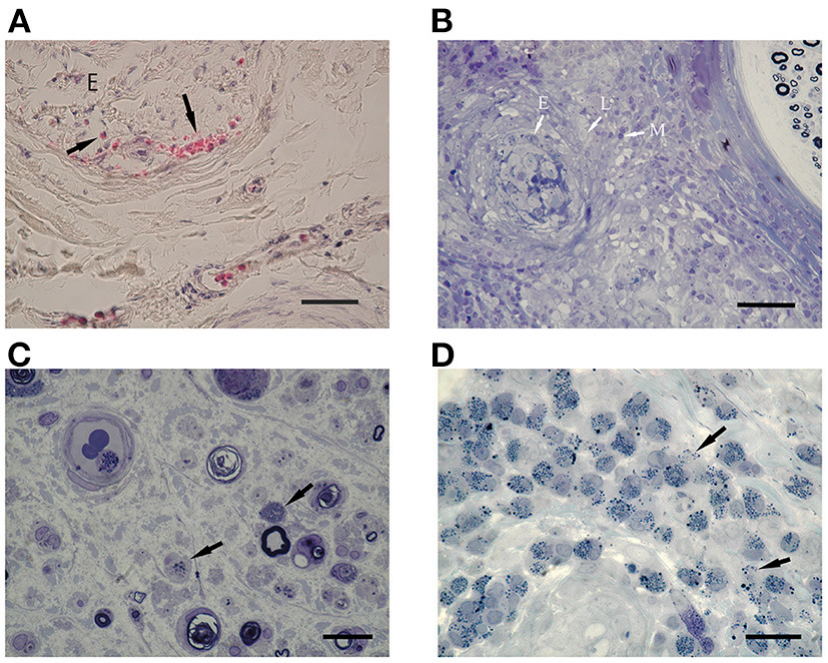
Eosinophilic infiltration. **(A)** HE staining shows endoneurial infiltration of eosinophils (arrows) in a patient with iHES. **(B)** In patients with ANCA-positive EGPA, infiltrates are comprised of mixed eosinophils (E) and other cells (L: lymphocytes, M: macrophages). **(C)** In a patient with ANCA-negative EGPA, endoneurial eosinophils show decreased cytoplasmic granules and piecemeal degranulation (arrows). Intraluminal eosinophils are also observed (arrowhead). **(D)** In ANCA-negative EGPA, eosinophils infiltrating the vessel wall show degranulation (arrows). **(B–D)** EPON-embedded sections with toluidine-blue staining. Scale bars: 50 μm **(A,B)** and 220 μm **(C,D)**. ANCA, anti-neutrophil cytoplasmic antibodies; e, endoneurium; EGPA, eosinophilic granulomatosis with polyangiitis; HE, hematoxylin and eosin; iHES, idiopathic hypereosinophilic syndrome.

Representative eosinophilic infiltration of each three group was shown in Figures 3A–D, respectively. Eosinophilic infiltration into the epineurium was observed in eight patients with iHES, whereas accumulated eosinophils (>50 eosinophils in the whole section) were found in 55% (five of nine iHES patients), although they were not present in the vessel walls (Figure 2A). Eosinophil accumulation in the epineurium was observed more frequently in the iHES and ANCA-negative EGPA groups than in the ANCA-positive EGPA group (*p* = 0.018, 0.033, respectively, Table 2). Accumulated infiltrates in the ANCA-negative EGPA group consisted of mostly eosinophils (Figure 3D); however, in the ANCA-positive EGPA group, the infiltrates comprised a mix of eosinophils, lymphocytes, and macrophages (Figure 3B).

Endoneurial extravascular eosinophils were present in all the groups (Figures 3A,C), with no significant difference in their frequency. Endoneurial eosinophilic degranulation was exclusively suggested in three patients with ANCA-negative EGPA (Figure 3C).

### Intraluminal eosinophils

Epineurial vessels occupied by eosinophils were observed in one HES case, four ANCA-positive EGPA cases, and 10 ANCA-negative EGPA cases. In each case, the percentage of number of epineurial occluded vessels to total number of epineurial vessels was calculated, and then the mean values were analyzed between each group.

In our results, no significant difference in the percentage of these epineurial vessels was found among the three groups (Table 2).

**Figure 4:**
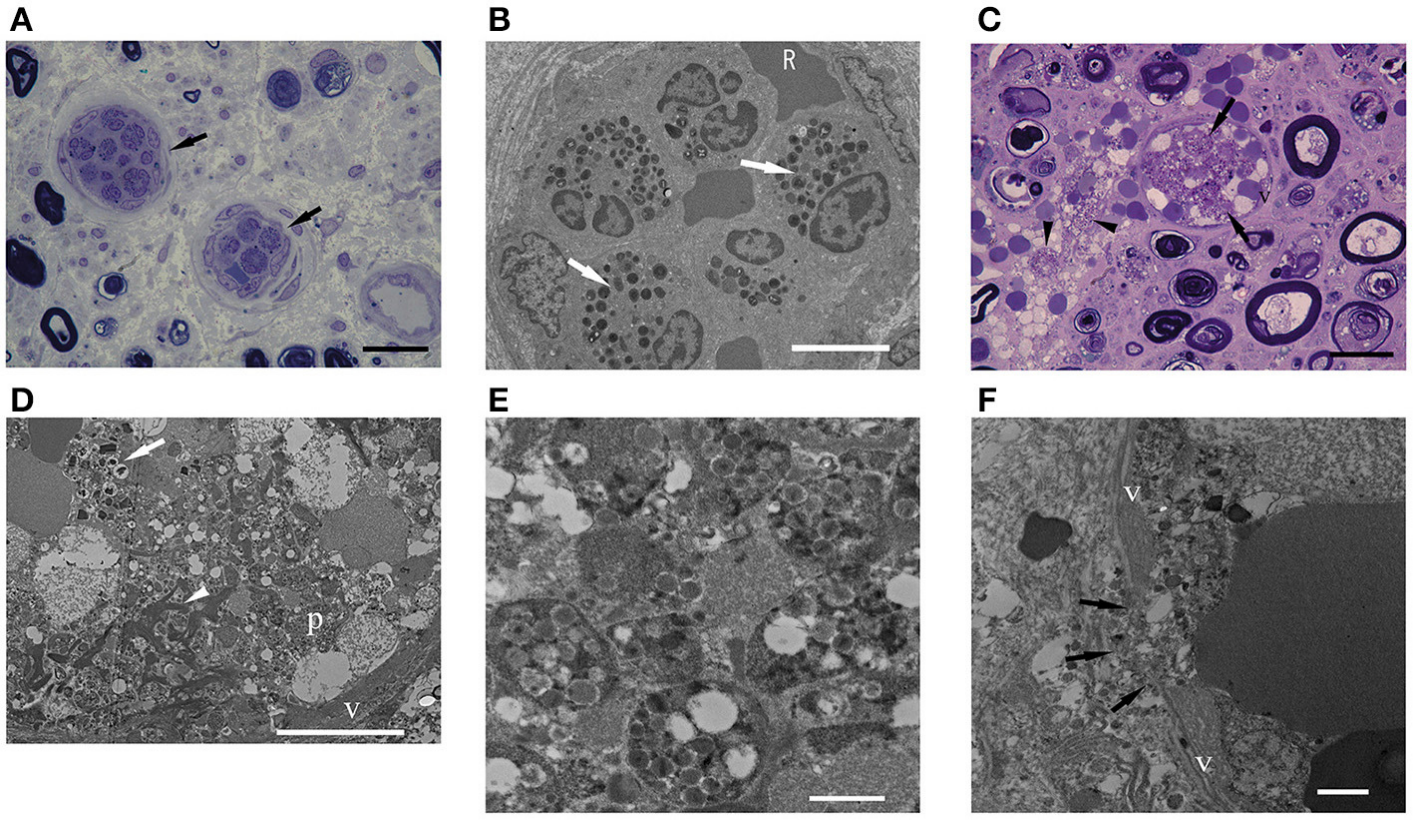
Intraluminal eosinophils. **(A,C)** EPON-embedded section with toluidine-blue staining. **(B,D–F)** Electron microscopy findings. **(A,B)** Intraluminal stenosis by accumulation of eosinophils in a patient with iHES. **(C–F)** ANCA-negative EGPA. **(A)** The percentages of intraluminal areas occupied by eosinophils: 60% (left arrow) and 62% (right arrow). **(B)** Electron microscopy showed that most of the vascular lumen was occluded by eosinophils and red blood cells. **(C)** Endoneurial vessel occluded by eosinophils (arrows). Infiltration of eosinophils into the extravascular space (arrowhead) and active axonal degeneration of myelinated nerve fibers is observed. **(D)** The occluded vessel in **(C)** occupied by eosinophils (arrow), fibrin (arrowhead), platelets (p), and red blood cells. Eosinophils showed characteristic crystalloid structures, although some granular contents were lost or swollen. **(E)** Platelet aggregates **(D)** are shown. **(F)** Endoneurial vessel destruction (arrows) by cytolytic eosinophil are observed in ANCA-negative EGPA. Scale bars: 20 μm **(A,C)**, 10 μm **(B,D)**, and 1 μm **(E,F)**. ANCA, anti-neutrophil cytoplasmic antibodies; EGPA, eosinophilic granulomatosis with polyangiitis; iHES, idiopathic hypereosinophilic syndrome; v, vascular endothelium.

Endoneurial vessels occupied by eosinophils were found in two iHES cases (Figure 4A) and two ANCA-negative EGPA cases, but not in ANCA-positive EGPA cases. The percentage of endoneurial vessels with over 50% of the lumen occupied by intraluminal eosinophils tended to be higher in the HES group than in ANCA-positive EGPA (*p* = 0.07) and ANCA-negative EGPA groups (*p* = 0.11), however, the difference was not statistically significant (Table 2). Electron microscopy in the iHES group showed that over 80% of intraluminal space was occupied by eosinophils and that the remaining space was packed with red blood cells (Figure 4B).

Most of the vessels occluded by eosinophils were small in diameter; however, one ANCA-negative EGPA case had endoneurial vessels that appeared swollen with red blood cells and eosinophils (Figure 4C). Electron microscopy showed that the eosinophils exhibited morphologies similar to extracellular traps in the lumen (26). Moreover, platelets, fibrinoid materials, and red blood cells were mixed with eosinophils, forming thrombi (Figures 4D,E). A portion of the endothelial layer was broken, through which lytic eosinophils appeared to infiltrate extravascular endoneurium (Figure 4F).

## Discussion

This is the first report to discuss the histological differences in peripheral nerves between patients with iHES and EGPA. iHES often involves peripheral neuropathy and shares common characteristics with EGPA. The neuropathic pattern of EGPA mostly involves mononeuritis multiplex due to the patchy distribution of ischemic lesions (8). In contrast, the current study showed that iHES exhibited symmetric progression of polyneuropathy, which is consistent with a previous report (27). This may explain the lower incidence of vasculitis in the iHES than in EGPA group. Vasculitis is one of the pathologies involving various organs associated with iHES, and is named eosinophilic vasculitis (18). Although 24% of patients with asthma-free eosinophilic vasculitis exhibited peripheral neuropathy, nerve biopsy-proven vasculitis has not been reported. In this study, true vasculitis was rare in the iHES group, which is in accordance with the preponderance of the phenotype of polyneuropathy. Thus, iHES-associated neuropathy may develop through additional mechanisms other than vasculitis. Extravascular eosinophils release of various toxic chemicals may cause symmetric damage.

Prevalence of necrotizing vasculitis in ANCA-positive EGPA, especially fibrinoid necrosis of vessel walls, has been previously described (8). In contrast, our study showed that epineurial infiltration of eosinophils was abundant in iHES and ANCA-negative EGPA. Epineurial vessel destruction was frequent in ANCA-negative EGPA but not in iHES, which relates to the preponderance of mononeuritis multiplex phenotype in ANCA-negative EGPA compared to iHES. iHES-associated neuropathy features non-vasculitic eosinophilic infiltrates, differentiating it from ANCA-negative EGPA.

In our study, three patients with secondary HES also showed polyneuropathic pattern; however two of the three showed fibrinoid necrosis histologically, distinct from iHES. This suggests that patients with HES and fibrinoid necrosis should be thoroughly screened for secondary causes of HES.

Eosinophil-associated vascular occlusion is often found in ANCA-negative EGPA (10). In this study, all groups showed intraluminal occlusion by eosinophils. Interestingly, the frequency of intraluminal occlusion in the epineurium tended to be higher in the ANCA-negative EGPA group than in the iHES, whereas in the endoneurium, it tended to be higher in the iHES group than in the ANCA-negative EGPA. Patients with iHES may develop eosinophil-associated vascular occlusion similar to those with EGPA in smaller endoneurium vessels, leading to nerve damage.

Eosinophils have multiple degranulation mechanisms, namely exocytosis, piecemeal degranulation, and cytolysis (28). Piecemeal degranulation is the selective secretion of various proteins, and the current study suggested this pattern in the endoneurium of EGPA (Figure 3C). The cytolysis of eosinophils has been referred to as eosinophil ETosis, which involves the release of filamentous chromatin structures called eosinophil extracellular traps. Our colleague had recently reported lytic eosinophils and ETosis in small-vessel thrombi in tissues from patients with EGPA (13). In one case with ANCA-negative EGPA, endoneurial vessels exhibited thrombi mixed with eosinophils, fibrinoid materials, and red blood cells. Furthermore, ETosis provide a scaffold for platelet adhesion (13). Similarly, eosinophils under cytolysis have been shown to break the endothelial barrier, promoting the intravascular occurrence of ETosis in patients with ANCA-negative EGPA and endoneurial bleeding. However, this has not been observed among those with iHES.

The current study suggested that in patients with iHES, eosinophils may cause neuropathy through the degranulation of toxic materials and endoneurial vascular occlusion, thereby causing nerve ischemia. Given that interleukin-5 is a key regulator of eosinophils, an anti-interleukin-5 monoclonal antibody has been used for the treatment of EGPA (29). The clinical efficacy of this anti-interleukin-5 monoclonal antibody had also been investigated in HES (30), with the results of the current study potentially forming the basis for the observed benefits. The limitations of the current study include the small sample size and retrospective design over a period of 22 years, potentially leading to an underpowered study. Thus, further research is needed to confirm our results.

## Data availability statement

The original contributions presented in the study are included in the article, further inquiries can be directed to the corresponding author.

## Ethics statement

The studies involving human participants were reviewed and approved by the Ethical Committee of the National Hospital Organization Minami Kyoto Hospital. The patients/participants provided their written informed consent to participate in this study.

## Author contributions

Study conception, design, and drafting the manuscript: HT and NO. Acquisition of data: TK and HT. Analysis and interpretation of data: KK and NO. All authors contributed to the article and approved the submitted version.

## Funding

This work was supported by the Ministry of Health, Labor, and Welfare.

## Conflict of interest

The authors declare that the research was conducted in the absence of any commercial or financial relationships that could be construed as a potential conflict of interest.

## Publisher's note

All claims expressed in this article are solely those of the authors and do not necessarily represent those of their affiliated organizations, or those of the publisher, the editors and the reviewers. Any product that may be evaluated in this article, or claim that may be made by its manufacturer, is not guaranteed or endorsed by the publisher.
